# Supplementary material for: Effects of early life stage exposure of largemouth bass to atrazine or a model estrogen (17α-ethinylestradiol)
Source: PeerJ. 2020 Oct 2;8:e9614. doi: 10.7717/peerj.9614 (PMC7537618; doi:10.7717/peerj.9614)
Supplement: Supplemental Information 1 — Summary of Mean (SE) of growth and sex ratio data measured in 80 dps largemouth bass juveniles that were successfully identified as either female or male. [file peerj-08-9614-s001.docx]

**Table S1. Growth and sex ratio data for all replicate tanks.** Summary of Mean (SE) of growth and sex ratio data measured in 80 dps largemouth bass juveniles that were successfully identified as either female or male.

|  |  | **n ___________** | | **Length (mm)________** | | **Weight (g)____________** | | **Sex Ratio_** |
| --- | --- | --- | --- | --- | --- | --- | --- | --- |
| **Treatment** | **Survival (%)** | **Female** | **Male** | **Female** | **Male** | **Female** | **Male** | **% Female** |
| **Control** | **58.4 (2.6)** | **57** | **52** | **54.2 (1.0)** | **54.1 (1.4)** | **1.70 (0.16)** | **1.62 (0.15)** | **52.5 (5.3)** |
| Tank 2 | 57.4 | 11 | 18 | 52.6 (1.7) | 55.4 (2.1) | 1.49 (0.19) | 1.93 (0.37) | 37.9 |
| Tank 7 | 65.8 | 17 | 12 | 55.9 (1.2) | 56.8 (1.1) | 1.75 (0.13) | 1.81 (0.11) | 58.6 |
| Tank 15 | 56.9 | 16 | 10 | 52.4 (1.3) | 50.4 (1.3) | 1.42 (0.12) | 1.28 (0.10) | 61.5 |
| Tank 20 | 53.5 | 13 | 12 | 55.8 (3.3) | 53.8 (0.9) | 2.13 (0.55) | 1.46 (0.10) | 52.0 |
|  |  |  |  |  |  |  |  |  |
| **1 ng/L EE2** | **55.6 (2.2)** | **42** | **61** | **60.3 (0.4)*** | **58.3 (1.4)** | **2.37 (0.15)*** | **1.95 (0.11)** | **40.3 (6.4)** |
| Tank 3 | 53.0 | 8 | 13 | 61.1 (1.9) | 55.3 (1.3) | 2.62 (0.30) | 1.87 (0.16) | 38.1 |
| Tank 8 | 61.8 | 13 | 14 | 61.0 (2.1) | 60.6 (1.9) | 2.41 (0.34) | 2.21 (0.20) | 48.1 |
| Tank 13 | 52.0 | 6 | 20 | 60.0 (5.1) | 56.5 (1.3) | 2.50 (0.87) | 1.70 (0.13) | 23.1^††^ |
| Tank 23 | 55.8 | 15 | 14 | 59.2 (1.3) | 60.8 (1.5) | 1.95 (0.18) | 2.02 (0.24) | 51.7 |
|  |  |  |  |  |  |  |  |  |
| **10 ng/L EE2** | **52.9 (1.4)** | **94** | **1** | **59.5 (2.3)*** | **64.0** | **1.98 (0.22)** | **1.93** | **99.0 (1.0)**** ^††^ |
| Tank 1 | 54.7 | 27 | - | 55.8 (1.5) | - | 1.72 (0.17) | - | 100.0^††^ |
| Tank 11 | 55.8 | 20 | - | 55.4 (1.1) | - | 1.49 (0.12) | - | 100.0^††^ |
| Tank 18 | 50.1 | 23 | - | 63.1 (1.7) | - | 2.25 (0.21) | - | 100.0^††^ |
| Tank 24 | 51.0 | 24 | 1 | 63.8 (1.3) | 64.0 | 2.45 (0.20) | 1.93 | 99.0^††^ |
|  |  |  |  |  |  |  |  |  |
| **1 µg/L ATR** | **54.0 (2.9)** | **44** | **59** | **55.8 (0.6)** | **57.1 (1.5)** | **1.68 (0.09)** | **1.48 (0.13)** | **42.7 (3.5)** |
| Tank 6 | 60.0 | 9 | 14 | 57.6 (1.7) | 61.7 (3.0) | 1.73 (0.19) | 1.82 (0.59) | 39.1 |
| Tank 12 | 57.0 | 10 | 18 | 55.5 (1.1) | 55.8 (1.9) | 1.51 (0.11) | 1.18 (0.32) | 35.7 |
| Tank 14 | 47.1 | 14 | 13 | 55.1 (1.3) | 55.3 (2.3) | 1.56 (0.12) | 1.46 (0.23) | 51.9 |
| Tank 19 | 51.6 | 11 | 14 | 54.8 (2.9) | 55.5 (1.7) | 1.91 (0.50) | 1.44 (0.28) | 44.0 |
|  |  |  |  |  |  |  |  |  |
| **10 µg/L ATR** | **58.2 (2.3)** | **38** | **56** | **58.8 (1.3)** | **56.8 (2.3)** | **2.15 (0.18)** | **1.80 (0.23)** | **39.7 (5.4)** |
| Tank 4 | 62.3 | 11 | 11 | 55.2 (2.1) | 51.0 (1.8) | 1.73 (0.20) | 1.34 (0.13) | 50.0 |
| Tank 9 | 58.2 | 5 | 14 | 60.8 (3.2) | 60.6 (3.0) | 2.31 (0.32) | 2.01 (0.44) | 26.3^†^ |
| Tank 16 | 51.6 | 9 | 16 | 58.3 (2.2) | 55.3 (1.7) | 2.01 (0.20) | 1.52 (0.19) | 36.0 |
| Tank 21 | 60.7 | 13 | 15 | 60.7 (2.3) | 60.3 (1.5) | 2.54 (0.45) | 2.33 (0.20) | 46.4 |
|  |  |  |  |  |  |  |  |  |
| **100 µg/L ATR** | **54.1 (2.3)** | **55** | **58** | **58.7 (1.1)** | **60.1 (1.2)** | **2.08 (0.17)** | **2.23 (0.16)** | **48.4 (4.6)** |
| Tank 5 | 56.6 | 15 | 17 | 55.9 (1.2) | 57.0 (1.5) | 1.71 (0.15) | 1.87 (0.16) | 46.9 |
| Tank 10 | 50.2 | 9 | 16 | 60.9 (1.9) | 59.8 (1.5) | 2.55 (0.27) | 2.29 (0.20) | 36.0 |
| Tank 17 | 59.2 | 16 | 13 | 59.5 (1.9) | 60.8 (1.8) | 2.03 (0.22) | 2.13 (0.22) | 55.2 |
| Tank 22 | 50.5 | 15 | 12 | 58.5 (1.5) | 62.8 (2.3) | 2.03 (0.15) | 2.61 (0.31) | 55.6 |

* = p < 0.05, ** = p < 0.01 for treatment means significantly different from control tested by Dunnett’s post hoc

^†^ = p < 0.05, ^††^ = p < 0.01 for sex ratios significantly different than expected 1:1 female:male tested by chi-square analysis
